# Optimizing recovery of frozen human peripheral blood mononuclear cells for flow cytometry

**DOI:** 10.1371/journal.pone.0187440

**Published:** 2017-11-01

**Authors:** Bo Langhoff Hønge, Mikkel Steen Petersen, Rikke Olesen, Bjarne Kuno Møller, Christian Erikstrup

**Affiliations:** 1 Department of Clinical Immunology, Aarhus University Hospital, Aarhus, Denmark; 2 Bandim Health Project, Indepth Network, Bissau, Guinea-Bissau; 3 Department of Infectious Diseases, Aarhus University Hospital, Aarhus, Denmark; Centro Cardiologico Monzino, ITALY

## Abstract

**Introduction:**

Live peripheral blood mononuclear cells (PBMCs) can be frozen and thawed for later analyses by adding and removing a cryoprotectant, such as dimethyl sulfoxide (DMSO). Laboratories across the world use various procedures, but published evidence of optimal thawing procedures is scarce.

**Materials and methods:**

PBMCs were separated from blood collected from healthy Danish blood donors, and stored at -80°C after adding of DMSO. The essential steps in the thawing procedure were modified and performance was evaluated by flow cytometry with respect to the percentage and total yield of viable PMBCs.

**Results:**

The best-performing washing medium was Roswell Park Memorial Institute (RPMI) 1640 at 37°C with 20% fetal bovine serum. When using 10 mL washing medium in a 15-mL Falcon tube, samples should be centrifuged for at least 10 minutes at 500 *g*. We failed to detect any differences between the tested methods of mixing PBMCs with washing medium. Likewise, neither the thawing duration nor centrifugation temperature (20°C and 37°C) had any effect. PBMCs could be incubated (rested) for up to eight hours in a 37°C 5% CO_2_ incubator without affecting cell counts, but incubating PBMCs for 16 hours significantly decreased viability and recovery. In general, high viability was not necessarily associated with high recovery.

**Conclusion:**

Changing the thawing procedure significantly impacted PBMC viability and live cell recovery. Evaluating both viability and live PBMC recovery are necessary to evaluate method performance. Investigation of differential loss of PBMC subtypes and phenotypic changes during thawing and incubation requires further evaluation.

## 1. Introduction

Analyses of human peripheral blood mononuclear cells (PBMCs) can be done on freshly collected blood samples or on cryopreserved samples. Analysis of freshly collected samples is usually preferred because the cryopreservation could affect functional and phenotypic properties of cells [1–7]. On the other hand, using cryopreserved PBMCs could minimize operator-dependent inter-assay [8] and inter-laboratory variation [9], which is evident in multi-center clinical trials with local sample collection [10,11].

The freezing / thawing procedure consists of several steps. Although dimethyl sulfoxide (DMSO) is often used as cryoprotectant, a high concentration of DMSO is toxic to human leukocytes [12,13]. Therefore, cells are usually washed as part of the thawing process to minimize the duration of DMSO exposure [14]. Each step in the thawing process can be performed in several ways. Thawing time, type and temperature of washing medium, method of mixing cells and washing medium, centrifugation and incubation conditions vary between laboratories, and are all parameters that could be adjusted to optimize yield [15].

Published studies and protocols vary with respect to the method used to thaw PBMCs, and several publications fail to describe the method used [16]. There is a general lack of evidence-based cell thawing procedures that ensure maximum cell recovery. In this study, we systematically evaluated different steps in the procedure of thawing human PBMCs using paired samples from healthy blood donors. Our outcome measurements consisted of PBMC viability and absolute count of live PBMCs (PBMC recovery).

## 2. Materials and methods

### 2.1 Blood donor enrollment

Peripheral venous blood samples were collected from voluntary blood donors at the blood bank in Aarhus, Denmark [17]. Each donor contributed two filled 4-mL heparin tubes, and further sample processing was initiated within two hours.

### 2.2 Isolation of PBMCs

First, blood samples were diluted with an equal volume of tris-buffered (pH 7.5) saline solution (BSS) (Ampliqon A/S, Odense, Denmark). The diluted blood was then carefully layered on top of a volume of Lymphoprep (Stemcell Technologies Inc., Vancouver, Canada) equal to the original volume of the blood sample. The solutions were centrifuged at 1,000 *g* for 20 min at 20°C for formation of the PBMC layer. Meanwhile, a 15-mL polypropylene conical centrifuge tube (Corning Science, Tamaulipas, Mexico) with 2 mL BSS was prepared for each donor. After centrifugation, sterile glass pipettes were used to aspirate the PBMCs, and cells were transferred to the 15-mL tubes with BSS. PBMCs were then centrifuged again at 500 *g* for 10 min at 20°C followed by aspiration of the supernatant. Subsequently, PBMCs were resuspended, mixed with 4 mL locally produced heat-inactivated human serum pool (HSP) at 4°C from donors with blood type AB, and stored for 10 min at 4°C.

### 2.3 Freezing procedure

For each donor, 4 mL cryopreservation medium was prepared, consisting of 3.2 mL RPMI 1640 without L-Glutamine (ThermoFisher Scientific Inc., Waltham, USA) and 0.8 mL DMSO (OriGen Biomedical, Austin, USA). Mixing was performed by dropwise addition of the 4°C cryopreservation medium to the PBMCs suspended in HSP. Thus, the final concentration of DMSO was 10%. For each donor, 1 mL cell suspension was added to each of 8 cryotubes, with each tube thus containing a number of PMBCs corresponding to that obtained from 1 mL whole blood. The cryotubes were then immediately placed directly in a rack in a -80°C freezer, where they were stored until thawing. Samples were stored for up to 2 weeks before thawing and cell counting.

### 2.4 Evaluation of thawing methods

For each evaluation, PBMC viability and live PBMC count were analyzed on paired donor samples; i.e., when an evaluation contained six variations of a step in the thawing process, then six cryotubes with frozen PBMCs from each donor were included. For each evaluation, one parameter in the thawing process was adjusted while all other parameters were kept constant. If nothing else is stated, the constant parameters consisted of partly thawed PBMCs with ice is still visible, using 20% S-RPMI (FBS) as thawing medium at 37°C, mixing PBMCs and thawing medium by pouring PBMCs into the washing medium, leaving the centrifuge at room temperature (20°C), centrifuging for 10 minutes at 500 *g*, and not incubating PBMCs before staining.

**Fig 1:**
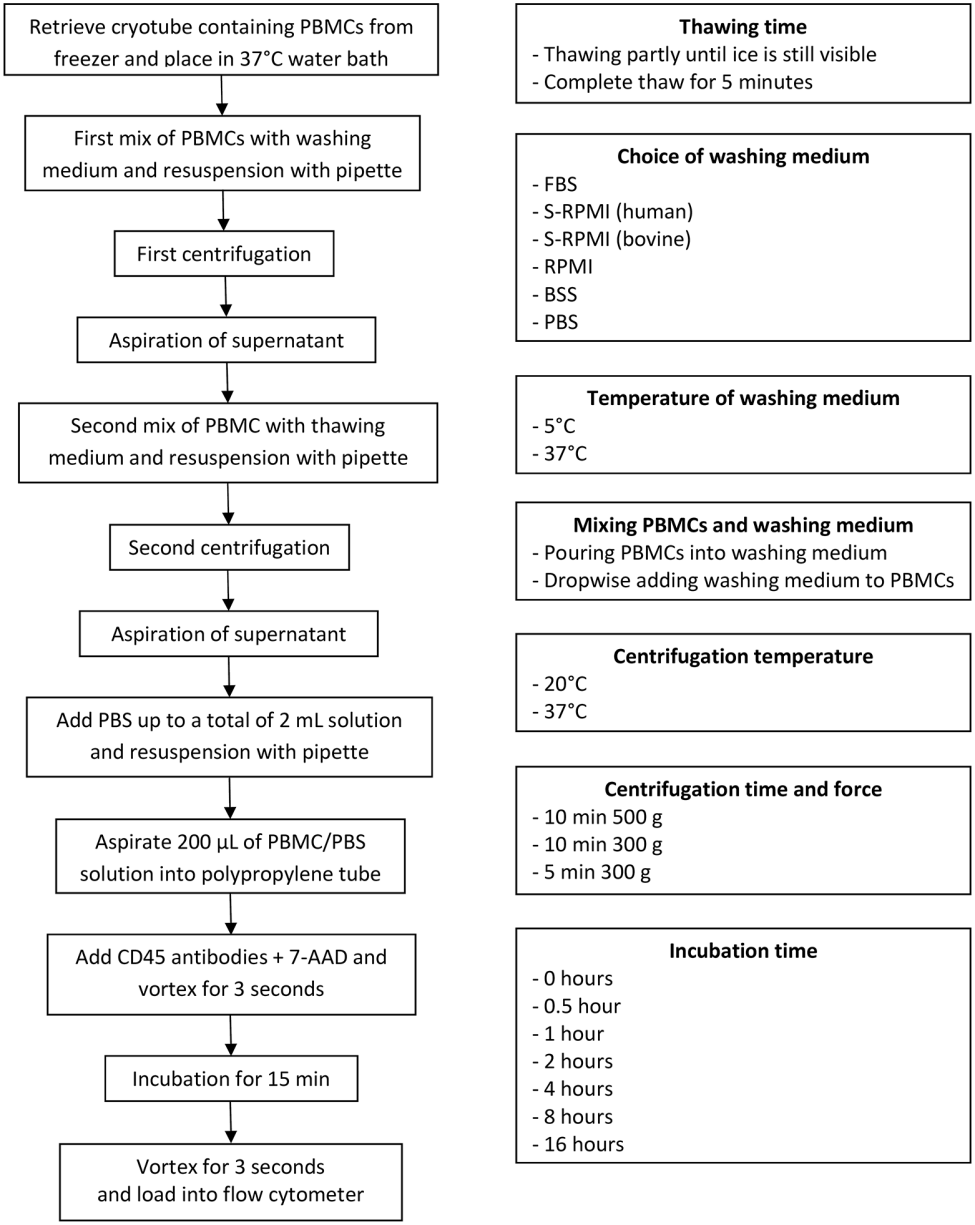
Flow chart of the thawing and staining process (on the left). Variations in the thawing procedure (on the right).

In all evaluations, the thawing process consisted of retrieving the PBMC-containing cryotubes from the -80°C freezer and placing them in a 37°C water bath for rapid thawing (Fig 1). The procedure involved two washing steps. First, using a Pasteur pipette the thawed PBMCs were mixed with 10 mL washing medium in a 15-mL polypropylene tube and centrifuged. The supernatant was then aspirated. Second, an additional 10 mL of washing medium was added, and the PBMCs were resuspended using a Pasteur pipette. The tube was then centrifuged once more, and the supernatant aspirated. The resuspended cells were then transferred to polypropylene tubes (Beckham-Coulter, 12x75 mm round bottom).

### 2.5 Flow cytometry

**Fig 2:**
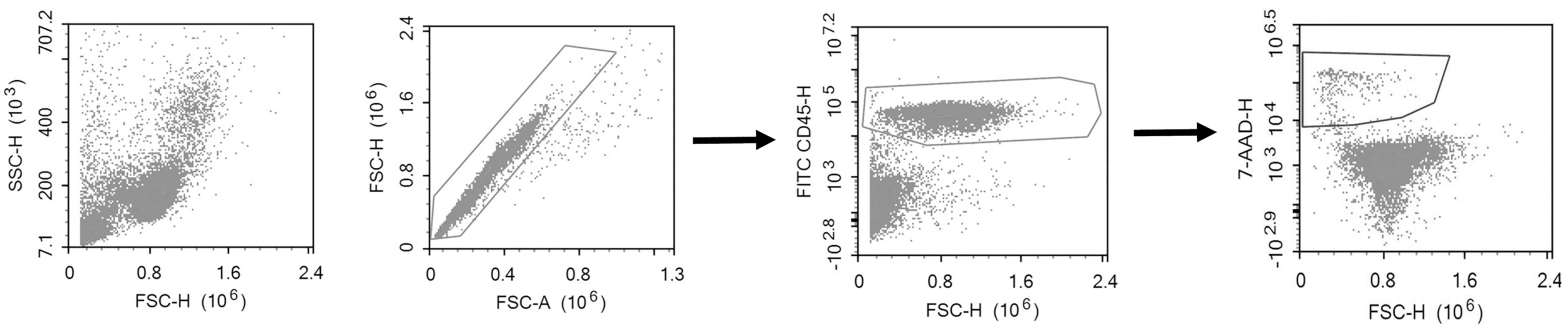
Gating strategy to identify live/dead CD45+ leukocytes. A) Events were triggered on FSC-H at a deliberately low threshold to avoid accidental exclusion of dead cells. B) Doublet exclusion C) Identification of CD45 positive cells D) Dead cells identified as 7-AAD positive events. Compensation for spectral overlap was not required.

After the two washing steps, PBMCs were resuspended in phosphate buffered saline (PBS). Samples were stained with 1 uL FITC-conjugated mouse-anti-human-CD45 antibody (BD, clone 2D1, Catalogue number 345808) for leukocyte count and 1 uL 7-AAD (1 mM) solution (Nordic BioSite, catalogue number ABD-17501) for viability staining. Absolute cell counting was performed using a volumetric NovoCyte flow cytometer (ACEA Biosciences, San Diego, USA). Gating was performed using NovoExpress 1.2.1 software (ACEA Biosciences, Inc., San Diego, USA), as visualized in Fig 2.

### 2.6 Statistical analyses

**Fig 3:**
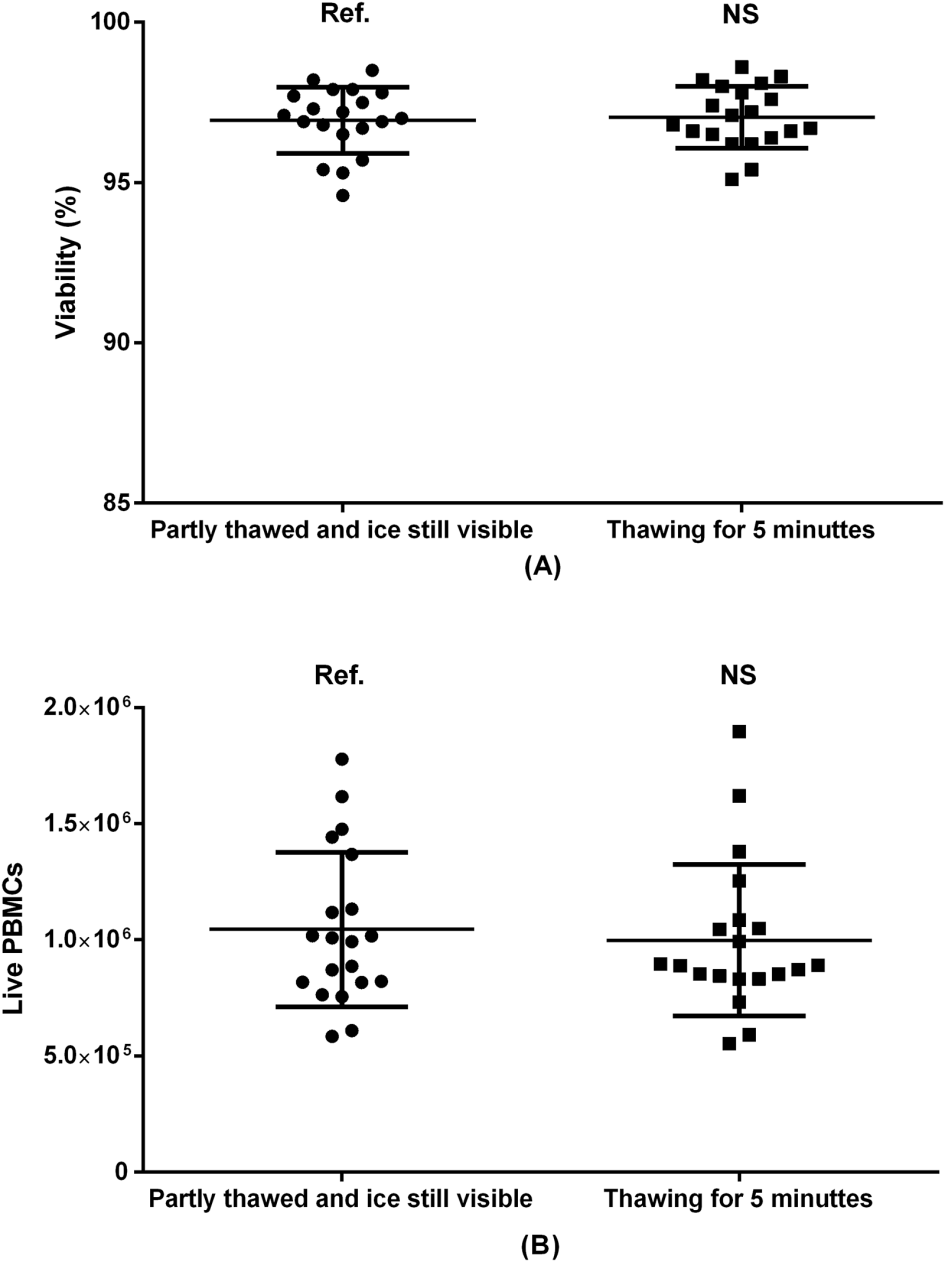
PBMC viability (A) and absolute recovery (B) as a result of varying thawing time in 37°C heated water bath. Paired samples from 20 donors included. Ref.: reference group. NS: non-significant.

**Fig 4:**
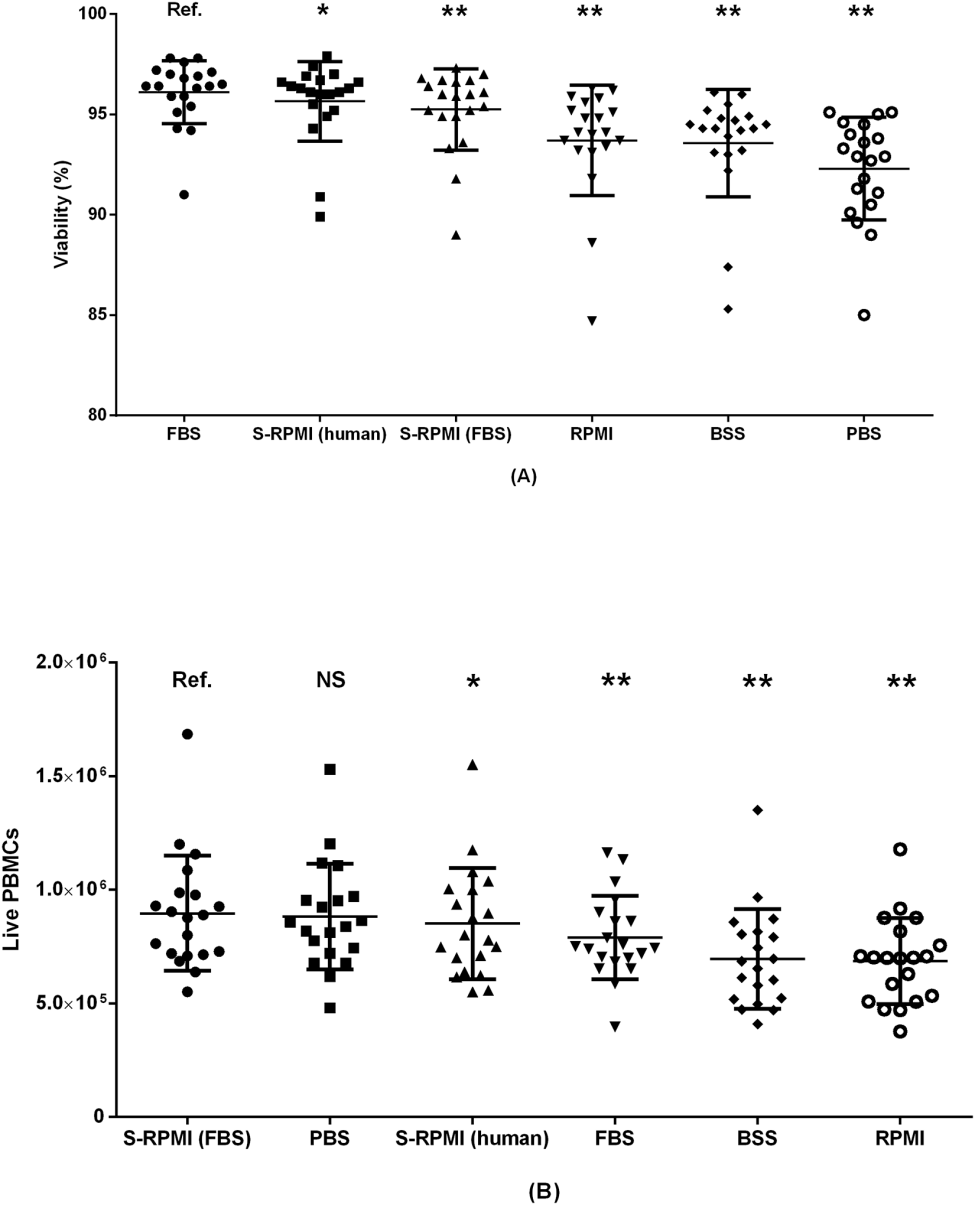
Performance of six different washing media presented in order of declining performance. Values presented with mean +/- SD. (A) PBMC viability (%) and (B) live PBMCs by thawing medium. Paired samples from 20 donors included. Ref.: reference group. NS: non-significant. *: p<0.05. **: p<0.01.

**Fig 5:**
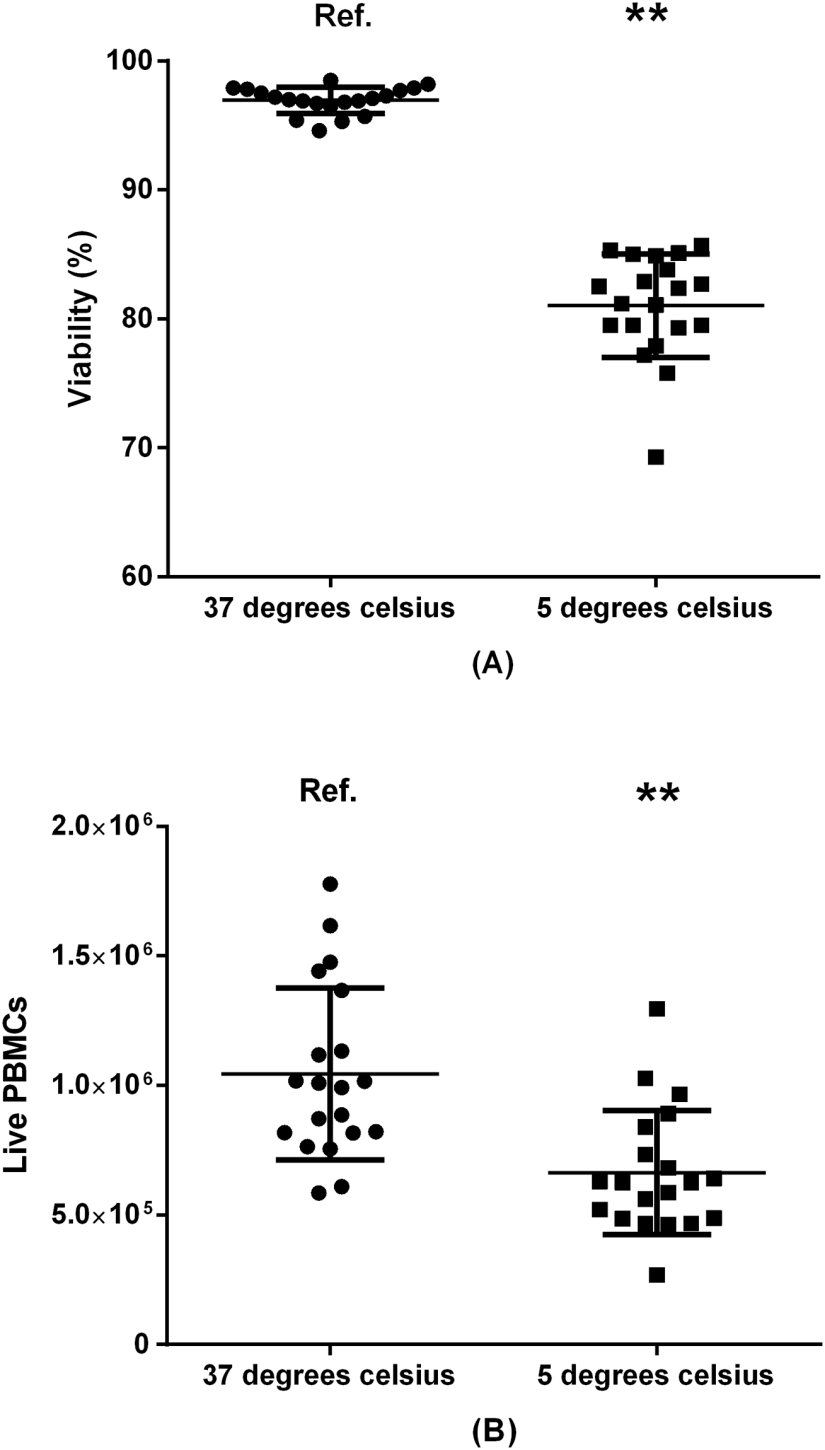
Impact of washing-medium temperature on viability (A) and PBMC recovery (B). 20 donors included. Ref.: reference group. **: p<0.01.

**Fig 6:**
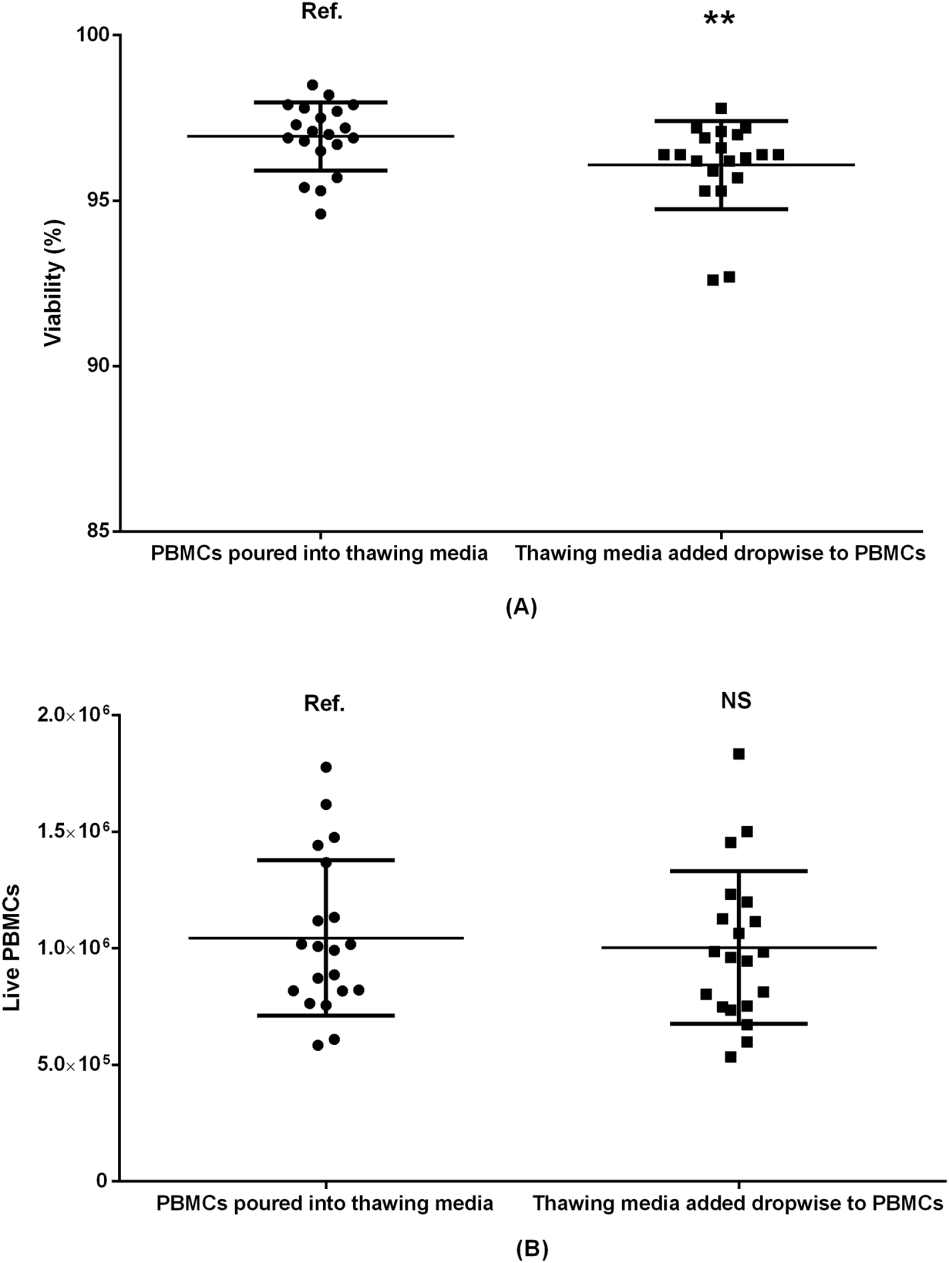
Comparing two ways of mixing PBMCs and washing medium by viability (A) and absolute count of live PBMCs (B). Paired samples 20 donors included. Ref.: reference group. NS: non-significant. **: p<0.01.

**Fig 7:**
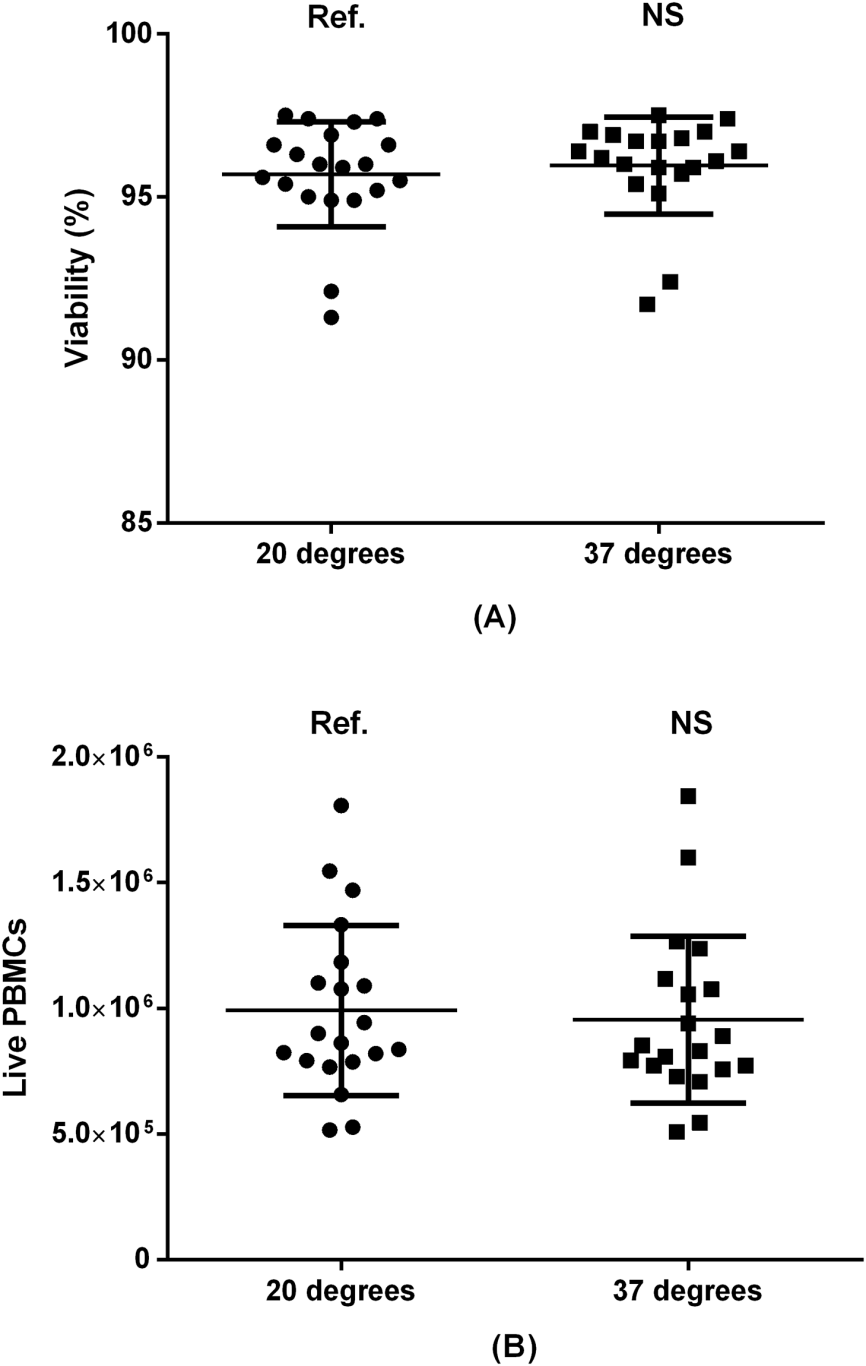
Temperature of centrifuge by PBMC viability (A) and absolute live PBMC recovery (B). Paired samples from 20 donors included. Ref.: reference group. NS: non-significant.

**Fig 8:**
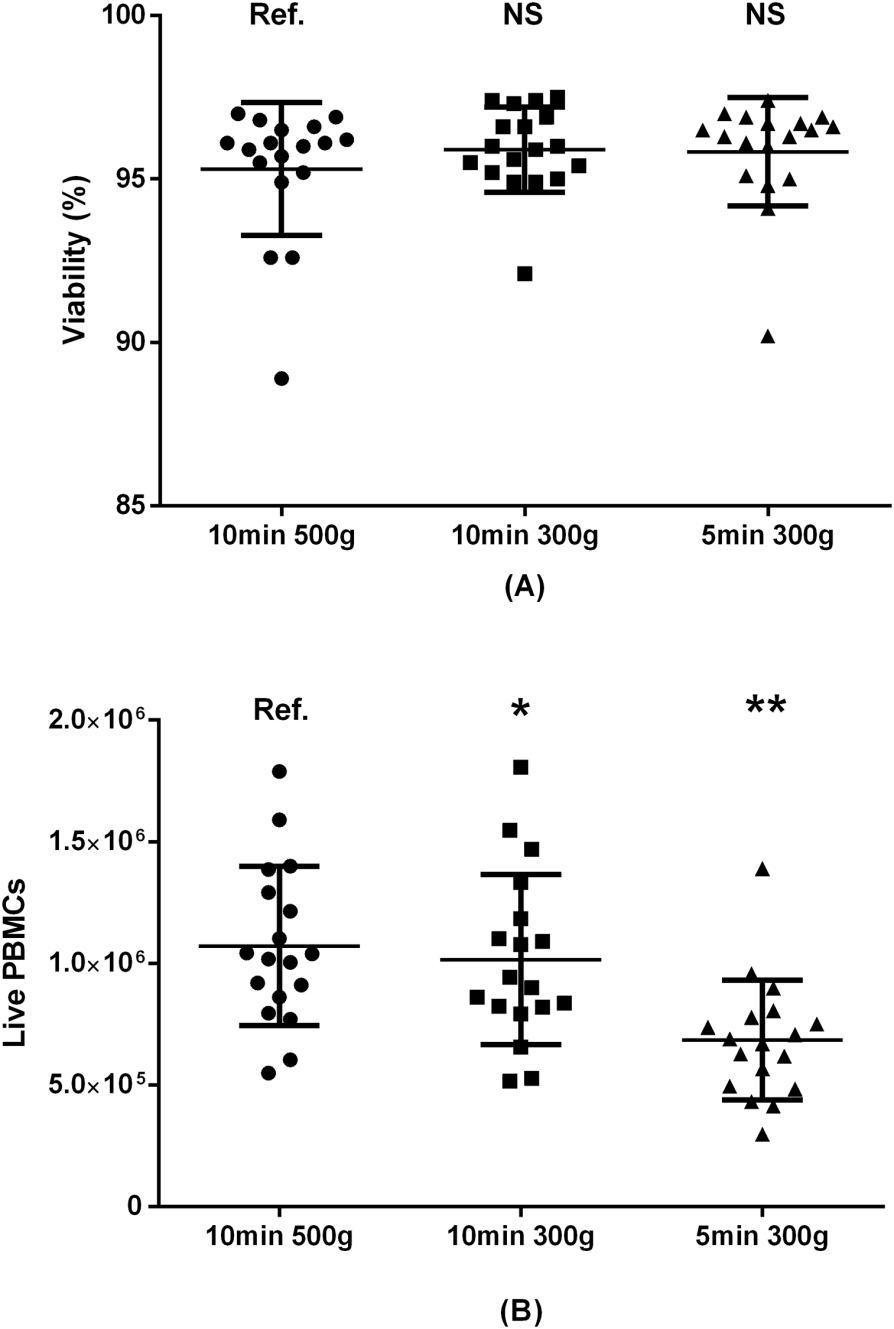
Centrifugation time and force and PBMC viability (A) and absolute live PBMC count (B). Paired samples from 18 donors included. Ref.: reference group. NS: non-significant. *: p<0.05. **: p<0.01.

**Fig 9:**
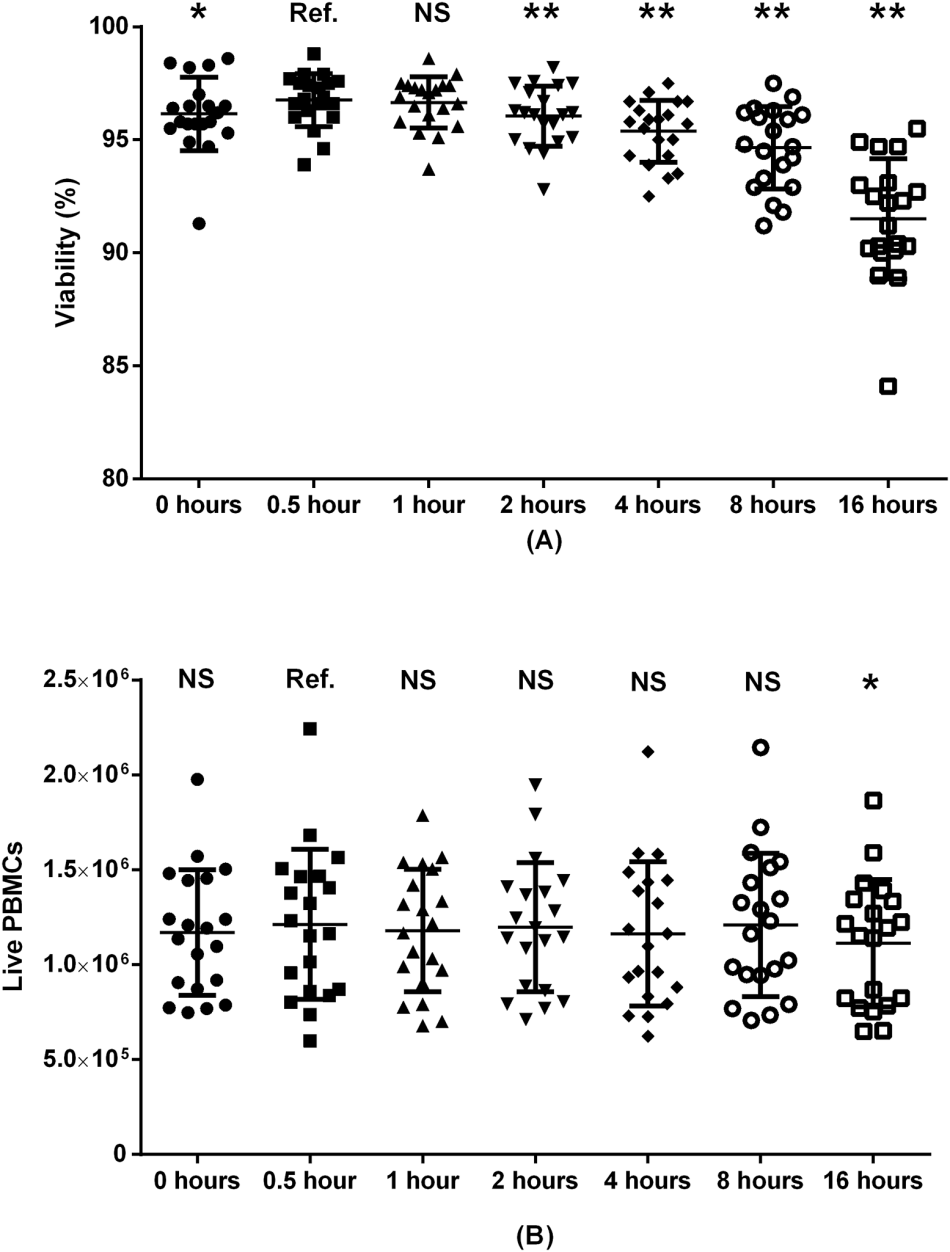
PBMC viability (A) and absolute live PBMC recovery (B) by incubation. Samples were stored in a 37°C incubator with 5% CO_2_. Paired samples from 20 donors were included. Ref.: reference group. NS: non-significant. *: p<0.05. **: p<0.01.

The best-performing method for each evaluation was identified by comparing paired means of the percentage of viable cells and by comparing paired absolute counts of live leukocytes. The thawing procedures were compared using paired t-test after ensuring normal distribution in Stata IC 13.0 (StataCorp, Texas, USA). Figs 3–9 were made using PRISM (Graphpad Software, CA, USA).

### 2.7 Ethics

Blood samples were collected anonymized from volunteer donors during routine blood donation as part of a quality assurance program (project number 119). According to Danish law, this does not require separate ethical approval.

## 3. Results

### 3.1 Thawing time

As previously suggested [16], cryotubes containing frozen PBMCs can be kept in a 37°C heated water bath until either the content is partly thawed and ice is still visible (approximately 1 minute and 15 seconds), or until the content of the cryotubes has reached 37°C for several minutes. We evaluated the thawing time in heated water bath by comparing partly thawed samples with samples kept in the water bath for 5 minutes. The choice of method affected neither viability nor absolute PBMC count (Fig 3).

### 3.2 Choice of washing medium

A total of six different washing media were evaluated; BSS, FBS, PBS, RPMI, S-RPMI (bovine) and S-RPMI (human). The washing medium producing highest mean PBMC viability was FBS (96.1%), followed closely by S-RPMI (human) (95.7%) and S-RPMI (bovine) (95.2%), Fig 4. However, the best performing washing medium in terms of live PBMC recovery was S-RPMI (bovine), which resulted in a significant higher live PBMC count than S-RPMI (human) (1.06 fold difference, p = 0.04), and the other media except PBS. This demonstrates that cell viability and absolute cell recovery are not necessarily associated. Indeed, a harsh thawing method could result in total disintegration of compromised cells to a degree where all “fragile” dead cells are no longer discernible as individual cells in flow cytometric analysis, and only the most robust, live cells “make it”. Such a method would have a high viability (inasmuch as no dead cells are left)–but a low yield.

### 3.3 Temperature of washing medium

In the first washing step, washing media (20% S-RPMI (bovine)) were either pre-heated to 37°C in a water bath, or pre-cooled in a 4°C refrigerator (Fig 5). Mean viability was higher for cells washed with 37°C medium (96.9%) compared to 4°C Medium (81.0%, p<0.01). Similarly live PBMC recovery was greater in the samples washed with 37°C medium (1.62 fold difference, p<0.01).

### 3.4 Mixing PBMCs and thawing medium

PBMCs and washing medium may be mixed in different ways; we compared swift pouring of partly thawed PMBCs directly into the washing medium, with dropwise addition of washing medium to the partly thawed PBMCs (Fig 6). Although the viability was marginally higher in the samples where the partly thawed PBMCs were poured into the washing medium (96.9%) than the samples where washing medium was added dropwise to PBMCs (96.1%, p = 0.01), there was no difference in the absolute count of live PBMCs (p = 0.17).

### 3.5 Centrifugation temperature

Because we had just demonstrated a significant positive effect of using warm washing medium, we hypothesized that heating the centrifuge could also improve PBMC viability and recovery. We therefore compared the effect of pre-heating the centrifuge to 37°C with leaving the centrifuge at room temperature (20°C). As presented in Fig 7, pre-heating the centrifuge did not affect outcomes significantly.

### 3.6 Centrifugation time and force

For this evaluation, PBMCs were resuspended in 10 mL washing medium consisting of S-RPMI (bovine) before the two rounds of centrifugation. There was no difference in average cell viability when comparing centrifugation time and force (Fig 8); 95.8% for 5 minutes centrifugation at 500 *g*, 95.9% for 10 minutes at 300 *g*, and 95.3% for 10 minutes at 500 *g*. Conversely, the absolute live PBMC count was significantly higher for a 10-minute centrifugation at 500 *g* than 10 minutes at 300 *g* (1.07 fold difference, p = 0.04) and 5 minutes at 300 *g* (1.60 fold difference, p<0.01).

### 3.7 Incubation time

After the second resuspension in washing media, we placed the PBMCs in an incubator at 37°C with 5% CO_2_ for different durations (Fig 9). For viability, we found a slight positive effect of incubating the samples for 30 minutes or 1 hour before the second centrifugation and staining. However, in terms of live PBMC count, there was no effect of incubation except a lower number of cells after 16 hours (1.09 fold difference, p = 0.02). This difference became non-significant (p = 0.10) after adjusting for multiple comparisons.

## 4. Discussion

We systematically evaluated seven steps in the process of thawing PBMCs by measuring cell viability and absolute live PBMC count. The live PBMC count depended significantly on the choice and temperature of washing medium, the centrifugation force, centrifugation duration, and on the incubation duration. Within the tested range, we found no effect of changing thawing time, method of mixing PBMCs and washing medium, or centrifugation temperature.

There is limited evidence-based recommendations for conducting the thawing of PBMCs for analyses by flow cytometry [15]. To fill this gap, we compared variations in several steps of the process. We evaluated the thawing step, during which PBMCs are placed in a heated water bath, but found no differences between thawing until ice was still visible, and thawing for a full 5 minutes. Our findings corroborate the findings reported by Ramachandran et al. In their study, cells were similarly thawed until only a small portion of the ice remained, or kept in the water bath for 10 minutes [16]. The investigators found no difference in cell viability when using pre-heated (37°C) washing medium, but when the wash medium was cooled, and mixed rapidly with PBMCs, the viability was lower. A lower cell viability was also described by Disis et al. [18] when using washing medium at 4°C. Thus, previous reports suggest the use of pre-heated, 37°C medium. Furthermore, we found that thawing PBMCs until only a small amount of ice remains produced results similar to those obtained when leaving the sample in a heated water bath for 5 minutes.

The choice of washing medium significantly affected our outcomes. In the study by Disis et al. [18] cell viability was higher when using FBS compared with human AB serum. We found highest viability using pure FBS, but the number of live PBMCs was higher in samples treated with S-RPMI (bovine). This illustrates that cell viability is not ideal as the primary endpoint when optimizing thawing procedures even though it was used as the primary endpoint in prior studies [13,16,18]. Owing to possible activation of T cells by xenoproteins, human serum from AB positive donors, or autologous serum, in washing media is recommended over FBS [15] but human serum is not as readily available. A general concern when using serum in washing media–human or bovine–is the risk of biologically significant batch-to-batch variations.

Studies using FBS as an ingredient in the washing medium have used a range of different concentrations. We tested 100% FBS, 20% FBS mixed with 80% RPMI, and 100% RPMI, and found the highest cell recovery using 20% FBS 80% RPMI. Others have used 100% RPMI [19,20], 10% FBS [3,7,9,13,21], 20% FBS [5], 30% FBS [22] or 50% FBS [23] mixed with RPMI. And some studies simply fail to mention the FBS concentration in the washing medium [24]. As FBS is relatively expensive compared to RPMI, a low FBS concentration would be preferable if performance is not compromised. This needs to be elucidated.

Because the temperature of the washing medium affected cell viability and live cell recovery, we hypothesized that increasing the centrifugation temperature to 37°C could improve results further; however, we failed to detect any such effect. Instead, lowering centrifugation force and time produced higher viability and lower live cell recovery. In a previous study [18] there was no difference in cell viability when adjusting centrifugation time (5 vs. 10 minutes) or force (280 vs. 450 rpm). Although cell viability is unaffected, adjusting these parameters could change the absolute live cell count. The purpose of centrifugation is to collect cells at the bottom of the tube allowing for aspiration of the supernatant without cell loss. The centrifugation time and force necessary to retain cells at the bottom of the tube may depend on the cell type and concentration, the type and volume of the washing medium, and the geometry of the centrifugation tube. The data we have generated in this study should therefore be considered specific for centrifugation of PBMCs in 10 mL S-RPMI (bovine) in a 15-mL Falcon tube. Using other settings may require separate evaluation.

Incubating, or “resting”, cells after thawing is a common practice when performing functional assays [2,25,26]. The rationale is that dead and dying cells are eliminated during the “rest period”, and only viable cells remain. For practical reasons, resting may also be performed before staining and analysis by flow cytometry [3,27]. However, there is limited evidence for the duration of this resting time in terms of optimizing cell recovery. We found no impact on live PBMC recovery when incubating cells for up to eight hours, but extending the period to 16 hours resulted in significantly lower cell counts. Our study was not designed to evaluate potential changes in cell phenotype during freezing/thawing or during incubation. Indeed, the functional capacity of cells changes during an “overnight resting” period [28], and both biased loss of certain cell types and changes in cell phenotypes could theoretically occur. This constitutes a limitation to our study.

This study did not evaluate freezing procedures, which also differ between laboratories. We used a standard DMSO concentration of 10% in the freezing medium, but other concentrations are described elsewhere [13,29]. After mixing the PBMC suspension with the freezing medium and dispensing the solution in cryotubes, we immediately placed the cryotubes directly in a rack in a -80°C freezer. Other laboratories have used devices to ensure a controlled freezing rate [30,31], and our freezing procedure may be a potential source of bias. Our samples were stored for up to 2 weeks in freezer -80°C freezer instead of moving samples to a liquid nitrogen container after 24 hours which is generally recommended. Whereas freezing temperature may affect PBMC functionality [32,33], there is little evidence to suggest that the PBMC phenotype is affected by freezing temperature. Variations in DMSO concentration, freezing procedure and storage time may have influence on which thawing procedure gives the highest PBMC viability and recovery.

## Conclusion

Laboratories world-wide use different approaches when freezing and thawing leukocytes. We here show that the thawing procedure has crucial impact on the recovery of live cells. The best-performing washing medium was S-RPMI (FBS), which should be pre-heated to 37°C before use. Centrifugation force and time may depend on the washing volume, but when using 10 mL 20% S-RPMI (bovine) in a 15 mL Falcon tube, samples should be centrifuged for at least 10 minutes at 500 *g*. Other conditions such as thawing time, method of mixing PBMCs and washing medium, or centrifugation temperature, seemed to have little or no influence on cell viability and recovery. Incubating PBMCs for up to eight hours without consequences on cell counts allows for practical variations in the laboratory set-up. Potential impact of the thawing procedure, and use of FBS containing xeno-proteins, on cell phenotype and functional characteristics needs clarification.

## Abbreviations

BSS: balanced saline solution
DMSO: dimethyl sulfoxide
FBS: fetal bovine serum
PBMC: peripheral blood mononuclear cell
PBS: phosphate buffered saline
RPMI: Roswell Park Memorial Institute
SD: standard deviation
S-RPMI (bovine): RPMI 1640 with 20% bovine serum
S-RPMI (human): RPMI 1640 with 20% human serum
